# Hepatic Encephalopathy in Cirrhotic Patients and Risk of Small Intestinal Bacterial Overgrowth: A Systematic Review and Meta-Analysis

**DOI:** 10.1155/2022/2469513

**Published:** 2022-10-18

**Authors:** Xin Feng, Xiaoqing Li, Xin Zhang, Weiqing Chen, Yin Tian, Qingqing Yang, Yingying Yang, Hui Pan, Zheng Jiang

**Affiliations:** ^1^Department of Gastroenterology, The People's Hospital of Yubei District of Chongqing City, Chongqing 401120, China; ^2^Department of Gastroenterology, The First Affiliated Hospital of Chongqing Medical University, Chongqing 400016, China; ^3^Department of Gastroenterology, Chongqing University Cancer Hospital, Chongqing 400030, China

## Abstract

**Background:**

Hepatic encephalopathy (HE) is a neurological and psychiatric syndrome. Recent evidence suggests that HE is not only a disease of the liver and brain but is also related to the gut. Small intestinal bacterial overgrowth (SIBO) is well known to be associated with cirrhosis, but the relationship between SIBO and HE is unclear. We conducted this comprehensive systematic review and meta-analysis to determine the association between SIBO and HE in cirrhotic patients.

**Methods:**

We conducted a comprehensive literature search of all studies on the association of SIBO and HE in cirrhotic patients using the PubMed and Embase electronic databases. Studies were screened, and relevant data were extracted and analysed. We calculated the number of cases of SIBO in patients with HE and controls. We then compared the prevalence of SIBO between the two groups to calculate the odds ratios (ORs) and 95% confidence intervals (CIs). Funnel plots were constructed to identify potential publication bias.

**Results:**

Six studies with 414 participants (219 HE patients and 195 controls) met the inclusion criteria. The prevalence of SIBO in cirrhotic patients with HE was significantly higher than that in those without HE. The combined OR was 4.43 (95% CI 1.73-11.32, *P* = 0.002). The heterogeneity was moderate (*I*^2^ = 66%), and the funnel plot suggested no significant publication bias. Subgroup analysis showed that the OR was 1.95 (95% CI 0.63–6.09) in studies using the lactulose breath test (LBT) and 7.60 (95% CI 3.50–16.50) in studies using the glucose breath test (GBT). The prevalence of SIBO in cirrhotic patients was also related to the severity of liver disease.

**Conclusions:**

Our meta-analysis identified a strong association between SIBO and HE, and the risk of SIBO was 4.43 times higher among cirrhotic patients with HE than among those without HE. SIBO could be a predisposing factor for the development of HE in cirrhotic patients. Therefore, the importance of SIBO should be emphasized in patients with HE.

## 1. Introduction

Hepatic encephalopathy (HE) is a neurological and psychiatric syndrome that occurs in patients with liver disease and is related to metabolic disorders of the body [1]. However, its pathogenesis has not been fully elucidated. The classic pathophysiological concept of HE is based on hepatic cell dysfunction or a portosystemic shunt (PSS), resulting in elevated blood and brain ammonia levels. These high ammonia levels produce a wide range of nonspecific neurological and psychiatric manifestations. The main clinical manifestations can range from reduced concentration, personality changes, and abnormal behaviour to consciousness disorders, coma, and death. Dyscalculia, disorientation, and asterixis are the characteristic manifestations of HE.

Small intestinal bacterial overgrowth (SIBO) is a manifestation of alterations in the intestinal flora. It is characterized by an increase in the number of bacteria and/or abnormal types of bacteria in the small intestinal tract [2]. Traditionally, SIBO has been considered to result from malabsorption associated with intestinal motility disorders. Recently, it has been found to be associated with a number of common diseases, such as cirrhosis [3], inflammatory bowel disease (IBD) [4], irritable bowel syndrome (IBS) [5], systemic sclerosis [6], chronic pancreatitis [7], and Parkinson's disease [8]. In recent years, the gut-liver-brain axis has attracted increased attention. SIBO is well known to be associated with cirrhosis, but the relationship between SIBO and HE is unclear. Here, we conducted this systematic review and meta-analysis to explore the association between SIBO and HE in cirrhotic patients.

## 2. Materials and Methods

### 2.1. Search Strategy

We conducted a comprehensive literature search of all studies on the association of SIBO and HE using the PubMed and Embase electronic databases from inception to May 2022. Language restrictions were not applied in the initial search. Additionally, reference lists of identified articles and published meta-analyses were searched to identify all relevant articles. The search terms included “small intestinal bacterial overgrowth”, “small bowel bacterial overgrowth”, “SIBO”, “SBBO”, “hepatic encephalopathy”, and “HE”.

### 2.2. Study Selection

The eligibility criteria for the studies included in the systematic review and meta-analysis were as follows: (1) cohort studies or cross-sectional studies examining the association between SIBO and HE; (2) valid methods used to assess HE, such as psychometric hepatic encephalopathy scoring (PHES) and critical flicker frequency (CFF) testing; (3) SIBO can be diagnosed by a jejunal aspirate culture (JAC) count ≥ 10^3^ colony forming units (CFU)/mL (gold standard) or a positive lactulose breath test (LBT) or glucose breath test (GBT) [2], so studies are diagnosed SIBO using the GBT, LBT, or JAC count; and (4) studies that compared the prevalence of SIBO in cirrhotic patients with HE versus cirrhotic patients without HE. The exclusion criteria were as follows: (1) case series, case reports, review articles, animal studies, and letters; (2) studies that did not investigate the association between SIBO and HE; (3) studies that reported unclear data; and (4) studies that provided duplicate data. We did not determine the cut-off values for a positive test when the positive criteria were clarified. Two authors independently excluded articles based on the eligibility criteria and exclusion criteria and then extracted the data. Any discrepancies were resolved in consultation with the third reviewer.

### 2.3. Data Extraction and Quality Assessment

We extracted the following data from the studies: the first author, country, year of the study, etiology, method of SIBO detection, SIBO diagnostic criteria, prevalence of SIBO in the two groups, HE diagnostic test, sex, mean age, and quality assessment. Our study meets the Preferred Reporting Items for Systematic Reviews and Meta-analysis statement (PRISMA) requirements [9]. The quality of the included studies was evaluated using the Newcastle-Ottawa scale (NOS) [10], which includes the selection of study groups, the comparability of the groups, and the determination of the outcome of interest, with a maximum score of 9 stars. Studies that scored ≥7 were considered high quality, while those that scored <7 were considered low quality.

### 2.4. Statistical Analysis

Review Manager (RevMan) version 5.3 was used to analyse the data. We calculated the number of cases of SIBO in patients with HE and controls and compared the prevalence of SIBO between the two groups to calculate the odds ratios (ORs) and 95% confidence intervals (CIs). A *P* value < 0.05 was considered statistically significant. Cochrane's test was used to assess heterogeneity among studies, and a value of *I*^2^ > 50% was considered to indicate substantial heterogeneity. The random-effects model was used with statistically significant heterogeneity; otherwise, the fixed-effects model was used. Funnel plots were constructed to identify potential publication bias.

## 3. Results

### 3.1. Search Results

**Figure 1:**
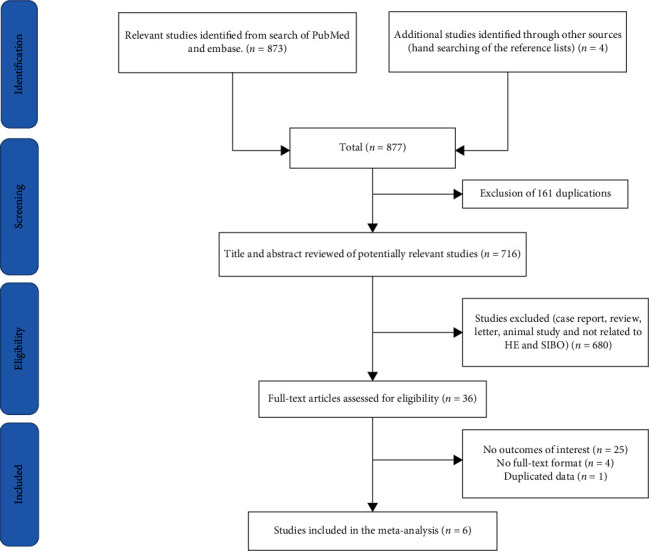
Flow chart of the selection process of the articles.

A total of 877 potentially relevant studies (533 from PubMed and 340 from Embase) were found in our search. Four studies were added after a manual search of the references. The titles and abstracts of 716 studies were reviewed after 161 duplicates were excluded. Subsequently, we excluded 680 studies that did not meet the inclusion criteria. After the full text was reviewed, 25 studies that had no outcomes of interest were excluded, and 4 studies were excluded because they were not full-text articles. One article was excluded because it shared duplicate data with another article. Finally, six studies [11–16] were included in this meta-analysis, including 414 participants (219 HE patients and 195 controls) (Figure 1). The characteristics and quality evaluation of the included studies are shown in Table 1. The studies mainly came from Asia and the America. These researchers used GBT or LBT to diagnose SIBO. The age and sex of the subjects in one study [11] were not disclosed, and it had a low-quality score.

### 3.2. Association between HE and SIBO

**Figure 2:**
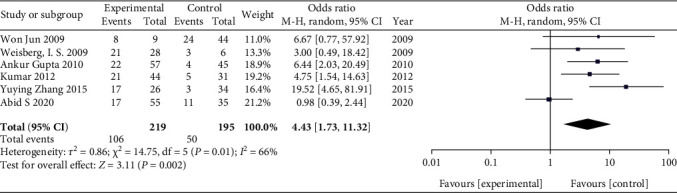
Forest plot of the odds ratios of SIBO in HE patients compared with controls.

**Figure 3:**
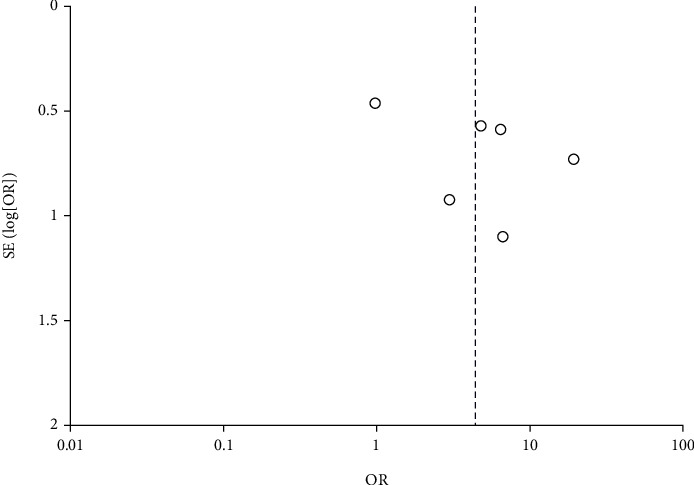
Funnel plot showing the odds ratios of publication bias in SIBO papers.

**Figure 4:**
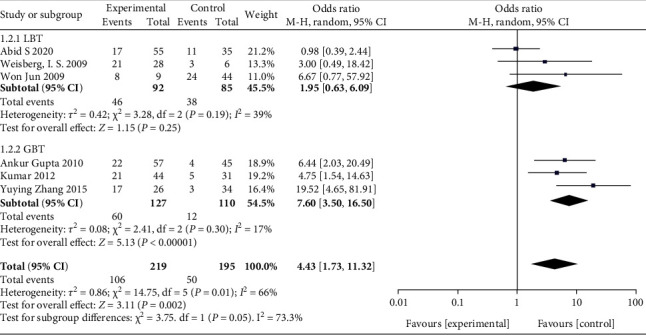
Forest plot of the odds ratios of SIBO based on the SIBO diagnostic test.

In this analysis, we compared the prevalence of SIBO in 219 HE patients and 195 controls. The prevalence of SIBO in cirrhotic patients with HE was significantly higher than that in cirrhotic patients without HE. The combined OR was 4.43 (95% CI 1.73-11.32), which was statistically significant (*P* = 0.002). Due to moderate heterogeneity (*I*^2^ = 66%), we used a random-effects model (Figure 2). A funnel plot was constructed based on effect estimates and the accuracy of each study to assess publication bias. The funnel plot suggested that no significant publication bias existed (Figure 3). Furthermore, in a subgroup analysis based on the SIBO diagnostic test used, the OR was 1.95 (95% CI 0.63–6.09) in three tstudies [11, 12, 16] using the LBT and 7.60 (95% CI 3.50–16.50) in three studies [13, 14, 15] using the GBT (Figure 4).

### 3.3. Association between Child-Pugh Class and SIBO

**Figure 5:**
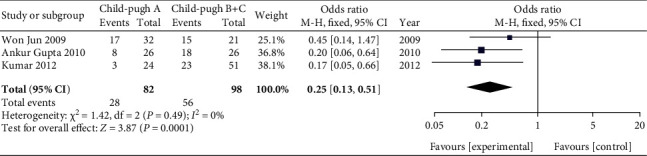
Forest plot of the odds ratios of SIBO in patients with Child-Pugh class A compared with patients with Child-Pugh classes B and C.

Three studies [12–14] in our meta-analysis showed an association between Child-Pugh class and SIBO in cirrhotic patients (Table 2). We compared the prevalence of SIBO in patients with Child-Pugh class A and patients with Child-Pugh classes B and C. We found that the prevalence of SIBO was lower in cirrhotic patients with Child-Pugh class A than in those with Child-Pugh classes B and C. The OR was 0.25 (95% CI 0.13-0.51), which was statistically significant (*P* = 0.0001) (Figure 5). These results indicated that the higher prevalence of SIBO in cirrhotic patients was related to the increased severity of liver disease.

## 4. Discussion

Due to increased research in recent years, the microbiome has been found to perform a wide variety of tasks in the human body. The gut microbiota plays a key role in the course of liver disease. Many studies have shown that the intestinal microbiota changes in cirrhosis patients, especially in patients with HE [17]. Liu et al. [18] found that the intestinal microecology of cirrhotic patients with minimal hepatic encephalopathy (MHE) was severely disproportionate, with significant overgrowth of potentially pathogenic Escherichia coli and Staphylococcus species. Bajaj et al. [19] found that the mucosal microbiome had a lower abundance of Roseburia and higher abundances of Enterococcus, Veillonella, Megasphaera, and Burkholderia in HE patients. Another study found that specific bacterial families (Alcaligenaceae, Porphyromonadaceae, and Enterobacteriaceae) are strongly associated with cognition and inflammation in HE patients [20]. The intestine and liver communicate through the portal vein, biliary tract, and systemic circulation. Intestinal products are transported to the liver through the portal vein and affect liver function. At the same time, the liver delivers bile acids through the biliary tract to the gut, which directly and indirectly inhibits bacterial overgrowth by regulating antimicrobial genes expression in host cells. Bile acids also protect the integrity of the small intestinal mucosa [21, 22]. In addition, the intestinal flora produces a variety of signalling molecules that can cross the blood-brain barrier and reach the central nervous system. Therefore, SIBO leads to disruption of intestinal motility and homeostasis, further leading to liver damage and affecting central nervous system function [23]. Gut-liver-brain axis dysfunction in cirrhotic patients may present as HE. The pathogenesis of HE is related to the imbalance of the intestinal microbiota and harmful microbial products (such as ammonia, indole, hydroxyindole and endotoxin) [24]. In HE patients, because of autonomic neuropathy and metabolic disorders, the orocecal transit time (OCTT) is delayed and SIBO is promoted. As cirrhosis progresses, portal hypertension leads to increased intestinal permeability and bacterial migration. The reduction of bile acid may promote bacterial overgrowth [25]. As SIBO exacerbates the OCTT delay and disrupts the intestinal barrier, more bacterial products flow into the liver, leading to the activation of liver immune cells and the release of proinflammatory cytokines. The diseased liver cannot inhibit SIBO and remove harmful microbial products effectively, thus accelerating disease progression and leading to HE [26].

JAC was considered the gold standard for the diagnosis of SIBO, despite its limitations such as invasiveness, high expense, difficulty to access the distal small intestine, and possibility of contamination by oral bacteria [2, 27–29]. Compared to JAC, breath tests (LBT and GBT) are noninvasive, inexpensive, and easy to be accepted by patients. The North American Consensus suggests that the standard doses of lactulose and glucose are 10 g and 75 g in breath tests [2]. The cut-off value for GBT was defined as an increase of ≥20 parts per million (ppm) above baseline in hydrogen within 90 minutes [2]. As for LBT, the cut-off value was considered as a rise of ≥10 ppm in methane [2]. A meta-analysis [30] has indicated that GBT has higher sensitivity and specificity than LBT. The results of LBT are susceptible to intestinal transport time. Lactulose may be transferred to colon quickly, because it is not absorbed in the small intestine. In this way, more lactulose may be degraded by colon bacteria and produce hydrogen rapidly, which will cause a false positive. In addition, patients with rapid intestinal transport will show an early peak that may be misinterpreted as SIBO.

This is the first systematic review and meta-analysis to investigate the prevalence of SIBO in HE patients. A pooled analysis showed that the risk of SIBO was more than four times higher in HE patients than in those without HE. This finding suggests that SIBO could be a predisposing factor for the development of HE in cirrhotic patients. The meta-analysis showed moderate heterogeneity in the studies. Moreover, we conducted a subgroup analysis based on the diagnostic test used for SIBO. We found that the risk of SIBO in studies using the GBT was higher than that in studies using the LBT. One possible explanation for this result is that glucose is rapidly absorbed earlier in the proximal small intestine than lactulose, causing a higher positive result rate. There are also discrepancies in diet, metabolism, and immune function among populations in different regions [31]. The prevalence of SIBO in patients with Child-Pugh class A and patients with Child-Pugh classes B and C was compared. The result showed that the prevalence of SIBO is closely related to the Child-Pugh classes in cirrhotic patients. With the severity of liver disease progress, the prevalence of SIBO increases. There are some limitations of our analysis. The sample size is small. Moreover, due to clinical operability, the included studies did not diagnose SIBO based on the gold standard (JAC) but instead used breath tests (LBT and GBT), which are more convenient and readily accepted by patients.

Based on the abovementioned information, a large part of the treatment for HE is the control of SIBO. Lactulose is the most widely used therapy. It can increase stool production, acidify the intestine, and alter the intestinal flora. This potential change in the microbiome may result in urease-producing bacteria being replaced by non-urease-producing Lactobacillus, thereby reducing the formation of potentially toxic short-chain fatty acids (such as propionate, butyrate, and valerate) [32]. Two studies in our meta-analysis investigated the effect of SIBO treatment on the clinical outcomes of HE patients. In the study by Abid et al. [16], MHE patients were treated with rifaximin (1200 mg/day for 1 week). After six weeks of follow-up, the presence of SIBO and the MHE status were reassessed. The overall improvement in MHE among patients with SIBO was statistically significant compared to those without SIBO. Zhang et al. [15] conducted a similar study by treating patients with rifaximin (200 mg, three times a day) orally for one week. SIBO and psychometric tests were repeated 4 weeks after antibiotic completion. A significant reduction in blood ammonia levels was observed in MHE patients with SIBO. Thirteen of 17 MHE patients with SIBO became SIBO negative, and their psychometric test scores also returned to normal. The results of the two studies [15, 16] demonstrated that treatment of SIBO with rifaximin can effectively improve MHE symptoms in patients with cirrhosis. It is speculated that rifaximin could potentially affect the brain by improving the microecology and autonomic nerve functions of the small intestine, thus improving cognitive ability. This study showed that rifaximin did not alter the relative abundance of intestinal bacteria but promoted a major shift in the complexity of the metabolome network [33]. *β*-Adrenoreceptor blockers speed intestinal transport and reduce intestinal permeability and bacterial translocation, thus reducing the incidence of SIBO [34, 35]. Probiotics, especially the lactose-fermenting action of Lactobacillus and Bifidobacteria, can improve the nutritional status of the intestinal epithelium, reduce intestinal permeability, and inhibit competition from pathogenic bacteria and can be used to treat HE [36, 37]. In addition, studies have discussed the therapeutic potential of faecal microbiota transplantation [38, 39].

## 5. Conclusion

Our meta-analysis identified a strong association between SIBO and HE, and the risk of SIBO was 4.43 times higher among cirrhotic patients with HE than among those without HE. SIBO could be a predisposing factor for the development of HE. Therefore, the importance of SIBO in HE patients should be emphasized. Additional multicentre and large sample studies are needed for further confirmation.

## Figures and Tables

**Table 1:** Main characteristics of the studies included in this meta-analysis.

Study	Country	Etiology	SIBO diagnostic test	SIBO diagnostic criteria	Prevalence of SIBO	Average age (years)	Male/female	Quality assessment
Weisberg et al. [11]	United States	HCV cirrhosis	LBT	10 g lactulose load is orally administered, (a) fasting breath H_2_ of >20 ppm, (b) increase in breath H_2_ in <90 min, (c) dual H_2_ peaks (12 ppm increase over baseline with decrease of 5 ppm before second peak), or (d) fasting breath CH_4_ of >10 ppm	Cases: 21/28 (75%) Controls: 3/6 (50%)	—	—	6
Jun et al. [12]	Korea	Cirrhosis	LBT	15 g lactulose load is orally administered, a basal hydrogen value of >20 ppm, or early hydrogen peak of ≥20 ppm (≥10 ppm in the case of methane gas) in the first 90 min	Cases: 8/9 (89%) Controls: 24/44 (55%)	55.1 ± 10.6	38/15	7
Gupta et al. [13]	India	Cirrhosis (alcohol, HBV, HCV, and others)	GBT	75 g glucose load is orally administered, rise of H_2_ ≥ 12 ppm over the baseline value within 2 hours	Cases: 22/57 (39%) Controls: 4/45 (9%)	Cases: 50.28 Controls: 44.9	Cases: 43/14 Controls: 41/4	7
Lunia et al. [14]	India	Cirrhosis (alcohol, HBV, HCV, and others)	GBT	100 g glucose load is orally administered, a rise of H_2_ ≥ 12 ppm over the baseline value within 3 hours	Cases: 21/44 (48%) Controls: 5/31 (16%)	Cases: 41.4 ± 9.11 Controls: 42.7 ± 10.7	Cases: 32/12 Controls: 23/8	8
Zhang et al. [15]	China	Cirrhosis (alcohol, HBV, and HCV)	GBT	A rise in breath hydrogen by 12 ppm above the basal level after glucose ingestion	Cases: 17/26 (65%) Controls: 3/34(9%)	48.9 ± 9.74	46/14	7
Abid et al. [16]	Pakistan	Cirrhosis (irrespective of cause)	LBT	50 g lactulose load is orally administered, rise of H_2_ ≥ 20 ppm over the baseline value within 90 min	Cases: 17/55 (31%) Controls: 11/35 (31%)	Cases: 44.6 ± 11.9 Controls: 45.5 ± 11.8	Cases: 29/26 Controls: 19/16	8

LBT: lactulose breath test; GBT: glucose breath test; ppm: parts per million; SIBO: small intestinal bacterial overgrowth.

**Table 2:** SIBO in various Child-Pugh class.

Study	SIBO in patients with child A	SIBO in patients with child B	SIBO in patients with child C
Jun et al. [12]	17/32 (53%)	9/13 (69%)	6/8 (75%)
Gupta et al. [13]	8/26 (31%)	18/26 (69%)	
Lunia et al. [14]	3/24 (13%)	13/31 (42%)	10/20 (50%)

## Data Availability

No data were used to support this study.
